# Revolutionizing Patient Monitoring in Age-Related Macular Degeneration: A Comparative Study on the Necessity and Efficiency of the AMD VIEWER

**DOI:** 10.3390/bioengineering10121426

**Published:** 2023-12-15

**Authors:** Hitoshi Tabuchi, Tomofusa Yamauchi, Toshihiko Nagasawa, Hodaka Deguchi, Mao Tanabe, Hayato Tanaka, Tsutomu Yasukawa

**Affiliations:** 1 Department of Technology and Design Thinking for Medicine, Hiroshima University, Hiroshima 734-0037, Japan; 2 Department of Ophthalmology, Tsukazaki Hospital, Himeji 671-1227, Japan; 3 Department of Ophthalmology, Nagoya City University, Nagoya 467-0001, Japan

**Keywords:** age-related macular degeneration, electronic medical record, information and communication technology, medical system, long-term follow up

## Abstract

(1) Background: Age-related Macular Degeneration (AMD) is a critical condition leading to blindness, necessitating lifelong clinic visits for management, albeit with existing challenges in monitoring its long-term progression. This study introduced and assessed an innovative tool, the AMD long-term Information Viewer (AMD VIEWER), designed to offer a comprehensive display of crucial medical data—including visual acuity, central retinal thickness, macular volume, vitreous injection treatment history, and Optical Coherent Tomography (OCT) images—across an individual eye’s entire treatment course. (2) Methods: By analyzing visit frequencies of patients with a history of invasive AMD treatment, a comparative examination between a Dropout group and an Active group underscored the clinical importance of regular visits, particularly highlighting better treatment outcomes and maintained visual acuity in the Active group. (3) Results: The efficiency of AMD VIEWER was proven by comparing it to manual data input by optometrists, showing significantly faster data display with no errors, unlike the time-consuming and error-prone manual entries. Furthermore, an elicited Net Promoter Score (NPS) of 70 from 10 ophthalmologists strongly endorsed AMD VIEWER’s practical utility. (4) Conclusions: This study underscores the importance of regular clinic visits for AMD patients. It suggests the AMD VIEWER as an effective tool for improving treatment data management and display.

## 1. Introduction

Electronic medical records (EMRs) are an essential medical development [1]. Reportedly, additional information improves the quality of decision making [2]. For example, in ophthalmology, especially in clinical glaucoma, progress graphs of visual field test results are often used in daily clinical practice [3].

However, using EMR systems specifically for the long-term management of age-related macular degeneration (AMD) has been rare, which, along with glaucoma, is a significant cause of midlife blindness [4]. The prognosis of AMD has improved with the advent of anti-vascular endothelial growth factor (VEGF) therapy [5]. AMD is an unstable disease requiring multiple invasive treatments due to the varying symptoms, repeated slowdowns, and worsening of the condition [6]. It is common for the same patient to receive a cumulative total of several dozen treatments [7]. Consequently, as numerous patients visit hospitals, medical information accumulates in large quantities. Both glaucoma and AMD, which require extended consultation periods, have high treatment dropout rates that interrupt medical care [8]. Distrust of physicians and treatment increases the dropout rate, and a lack of explanation of medical conditions amplifies patient anxiety and doubt [9].

From the perspective of avoiding dropouts, it is essential to measure macular volume and central retinal thickness, which correlate with the activity of AMD, and to provide continuity of explanation to patients [10,11]. Nevertheless, no EMR tool can compile a list of treatment event dates and visual and anatomical information, which is particularly important for supporting the acquisition and understanding of AMD-specific information. The importance of such an EMR tool for the long-term management of AMD is heightened due to the significant impact that information on treatment intervention dates can have on physician decision making.

Therefore, we developed a system (the AMD Long-term Information Viewer, AMD VIEWER) that displays vital clinical AMD data. Those data include corrected visual acuity, macular volume, central retinal thickness, history of invasive treatments such as anti-VEGF injections, and optical coherence tomography (OCT) imaging information in chronological order. The purpose of AMD VIEWER is to provide a new, practical clinical resource that enables physicians and patients to obtain a complete picture of a large amount of long-term medical information on AMD in daily medical examinations.

This study aimed to clarify the necessity and efficiency of AMD VIEWER. To support the need for AMD VIEWER, we surveyed AMD patients with a history of treatment, such as photodynamic therapy (PDT) and vitreous injection, including the frequency of visits and the number of dropouts. In addition, to demonstrate the efficiency of AMD VIEWER, we measured the time needed for the system to display information. We compared it to the time humans need to retrieve and input the same amount of information. The usefulness of AMD viewer in clinical settings was evaluated by obtaining Net Promoter Scores (NPS) [12,13] (whether they would recommend its use to their close ophthalmologist colleagues) from ophthalmologists who have experience using it.

## 2. Materials and Methods

### The AMD VIEWER

***System***

**Figure 1:**
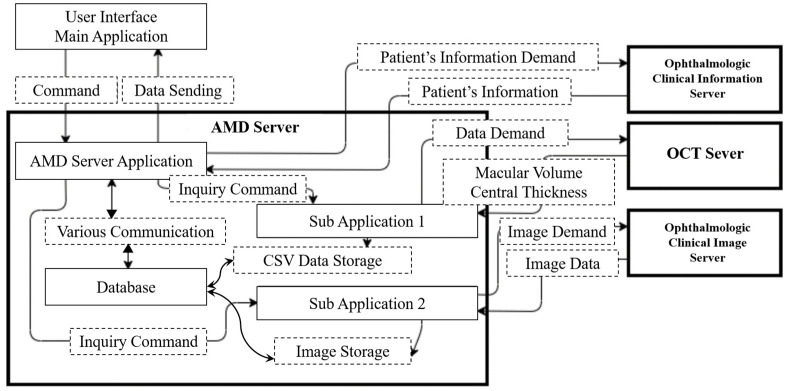
The system configuration diagram.

Figure 1 shows the system configuration diagram. The AMD VIEWER system consists of a user interface main application (Main App) on a client PC and four servers connected by a hospital local area network (LAN). The servers comprise an AMD server, an ophthalmology information server, an OCT server, and an ophthalmology image server. Triggered by input instructions from the Main App, two original collaborative applications placed on the AMD server automatically retrieve necessary information from the other three servers and display it on the interface monitor. All collected data are stored in the AMD server’s internal database. From the subsequent access, a combination of stored and newly acquired data is displayed on the monitor (technical details regarding the server cannot be provided for security reasons).

***Main Application (Main App)***

**Figure 2:**
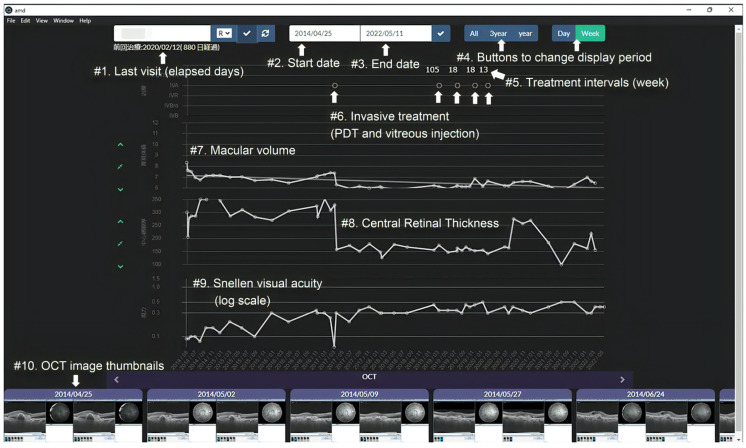
Example of a monitor display for AMD VIEWER.

Figure 2 shows an example of a monitor display for AMD VIEWER. One monitor displays lists of medical data for one patient’s left and right eye, including the history of treatment and ophthalmic clinical data. The treatment history includes information on four anti-VEGF injections and other treatment modalities: ranibizumab, aflibercept, brolucizumab, bevacizumab, steroid injections, and PDT. Ophthalmic clinical data have OCT macular volume, OCT central retinal thickness, and corrected visual acuity in time series during the examination period. These indicators were selected because they are clinically useful based on T.Y’s over 20 years of extensive experience treating AMD patients and can be automatically calculated within OCT device applications. It is possible to draw a regression line on these three values. The monitor also displays optical coherence tomograms taken on each examination date as thumbnails in chronological order. Users can enlarge the OCT images by clicking on the thumbnails. Supplemental Materials: Video S1 (THE AMD VIEWER DEMO) is a video recording of mouse operations and monitor behaviors for AMD VIEWER to display an individual’s data with AMD. This system was designed exclusively for patients who have been diagnosed with AMD and are under observation. It is not intended for use with patients who have cataracts or glaucoma. The assumption is that any changes in the patient’s visual function are attributable to AMD.

***OCT macular volume values***

We defined the OCT macular volume as the volume estimated from 64 slices within a circular area with a radius of 6 mm from the central macular fossa acquired with swept-source OCT (DRI Triton, Topcon, Japan), ranging from the retinal pigmentary epithelium (PRE) line to the inner limiting membrane. Using a linked application, the Main App displays these values graphically from the OCT server.

***OCT central retinal thickness***

We defined the OCT central retinal thickness as the distance from the PRE line to the inner limiting membrane within a 1 mm radius of the central macular fovea acquired with swept-source OCT (DRI Triton, Topcon, Japan). Using a linked application, the Main App graphically displays these values from the OCT server.

***Corrected 5-m visual acuity value***

The Main App displays Snellen visual acuity values in the logarithm of the minimum angle of resolution (logMAR) as extracted from the ophthalmology information server.

***OCT images***

The Main App displays a time series of the thumbnails of OCT images stored on the ophthalmic image server. The OCT images can be enlarged and displayed when the user clicks the corresponding thumbnail. Selecting OCT images is from those stored in an image filing server. The image filing system contains files tagged with an ID, date, and labels for the left and right eyes. Although the internal database of the OCT imaging device stores all captured images, a technician selects one horizontal and one vertical cross-sectional image from these, which are then stored in the image filing system by the date of capture. AMD VIEWER picks up OCT images from this filing system. After being captured by the AMD viewer, the OCT images and other information are logged and stored in a specialized database designed for the AMD VIEWER. This setup streamlines access to each database during future consultations. The data management process within the AMD Server’s Database involves selectively accumulating only those records with dates absent in the existing CSV Data Storage and Image Storage folders. Each new ID prompts the gathering and updating of these data within the server (Figure 1). So, the time required to switch between images of the left and right eyes is minimal. On new consultation dates, a program is designed to retrieve newly stored OCT images from that day in the image filing system.

**A comparative of the clinic attendance frequency and minimum visual acuity in active and dropout groups of AMD patients**

This study involved patients diagnosed with AMD who had undergone PDT or intravitreal injection therapy and were registered in the medical retina database of the Department of Ophthalmology at Tsukazaki Hospital. The parameters investigated included the duration and frequency of clinic visits, the number of intravitreal anti-VEGF injections received, and the dropout rate. A dropout was defined as a patient who either did not attend the clinic one month post appointment or had canceled the appointment. The dropout rate was calculated as the ratio of the number of dropouts to the total of active patients and dropouts. Active patients were defined as those continuing their visits to the hospital, excluding those who had died or been referred to other hospitals. Using Tukey’s HSD test, we compared the number of visits and treatments—comprising intravitreal anti-VEGF injections or PDT therapy—between the dropout and active groups. Furthermore, we used the Steel–Dwass test to compare the minimum corrected visual acuity (LogMAR conversion value of Snellen acuity) between the two groups during the observation period. For unilateral AMD, the eye with the condition was used for comparison, whereas in bilateral AMD, the eye with the poorest corrected visual acuity was considered.

**Comparing time requirements and error rates between manual data entry and AMD VIEWER monitor display**

**Figure 3:**
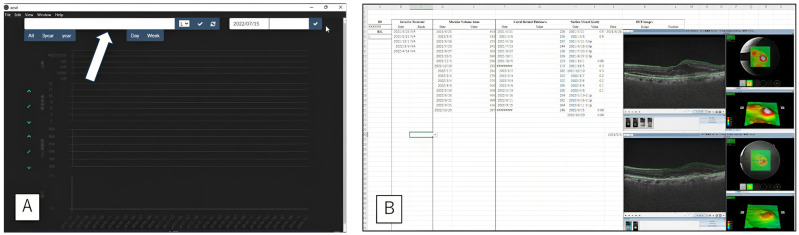
Screenshots of the AMD VIEWER and Manual Input Interface. (**A**) AMD VIEWER System (Note: the white arrow points to the ID input box.) (**B**) User Interface for Manual Data Entry (manually enter the necessary numbers into the Excel spreadsheet and paste the images without resizing them using copy and paste).

We examined the time it took for three optometrists to manually gather and input all medical data into a template sheet explicitly prepared for this study. The medical data comprised nine elements: ID, designation of left or right eye, date of examination, corrected visual acuity, macular volume as measured by OCT, central retinal thickness as measured by OCT, type and date of injection, and OCT images. For this study, a total of five eyes from five patients, with one, 10, 30, 50, and 100 visits, were included. The required medical information for 1, 10, 20, 50, and 100 consultations, including numerical data inputs, necessary OCT image pasting, and their total item counts were, respectively, (3, 1, complete 4), (27, 8, full 35), (57, 17, total 74), (164, 58, total 212), and (248, 75, total 323). The optometrist orthoptist responsible for the experimental data collection was briefed by the authors about the purpose and understands that the data collection is from the same patient. The data is retrieved by entering the ID for each stored server. We measured the AMD VIEWER monitor rendering speed (AMD VIEWER Time) for the same five patients and the averaged AMD VIEWER Time from ID entry to data display at three times: busy, quiet, and closed. (Figure 3) We performed multiple comparison tests (Tukey’s HSD test for Human Time Steel–Dwass test for AMD VIEWER Time) based on the number of hospital visits. We estimated the Human Time at 200 visits by plotting the linear regression line for the mean Human Time of the three optometrists.

**Evaluating the Usability of AMD VIEWER by Net Promoter Score (NPS)**

In this study, we collected the Net Promoter Score (NPS) from ten ophthalmologists with experience using AMD VIEWER to evaluate its usability. The NPS was originally a metric used in the business industry to measure customer loyalty. For this study, we slightly modified the question to fit our system, asking, “To what extent would you recommend this system to a close colleague in ophthalmology? Please rate it on a scale from 0 to 10, with ten being the highest”. To calculate the NPS, respondents are divided into three groups based on their scores: those who score 10–9 points are ‘Promoters’, those who score 8–7 points are ‘Passives’, and those who score 6–0 points are ‘Detractors’. The NPS is then a number from −100 to 100, calculated by subtracting the percentage of Detractors from the percentage of Promoters.

**Statistical calculations**

All statistical calculations were performed using JMP Pro 16.2.0 (SAS Institute, Cary, NC, USA).

## 3. Results

### 3.1. AMD Patients

**Figure 4:**
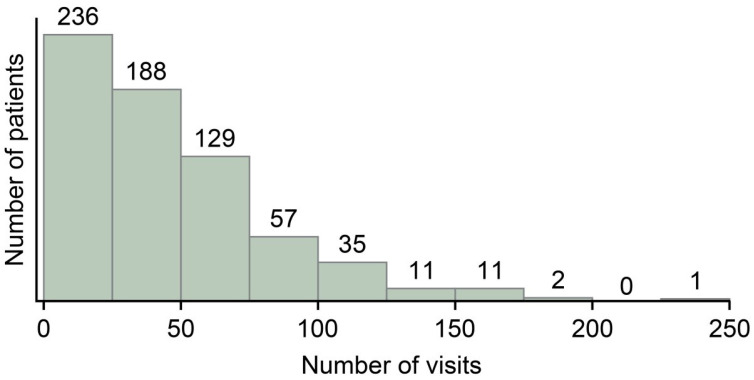
The frequency distribution table.

Of the database’s 977 AMD patients, we included 670 (68.6%) patients with PDT or vitreous injection therapy medical histories. The mean (SD) age of the subjects was 78.1 (9.2) years, and 492 (73.4%) were male. The mean (SD) interval from the earliest to the latest visit was 1934 (1457) (range 0–6557) days, and the mean number of visits was 45.7 (35.5) (range 2–225). Figure 4 shows the frequency distribution table. The mean number of vitreous injections was 10.3 (12.6). A total of 315 patients dropped out (dropout rate = 47.5%), 347 remained active, and 8 experienced other events (4 deaths and 4 referrals). The mean number of hospital visits for the dropout and functional groups was 42.9 (35.4) and 48.4 (35.7), respectively, significantly less in the dropout group (*p* = 0.0491 by Tukey’s HSD test). The number of injections was significantly lower in the dropout group (7.9, SD 9.1) than in the active group (12.6, SD 14.8) (*p* < 0.0001 by Tukey’s HSD test). Furthermore, the minimum corrected logMAR visual acuity value (maximum corrected Snellen visual acuity) during the observation period was 0.890 (±0.762) in the active group and 1.110 (±0.782) in the dropout group, with the active group showing significantly better results (*p* < 0.0001, Steel–Dwass test).

The details of each group and the demographic data are provided in Table 1.

### 3.2. Efficiency of AMD VIEWER

**Figure 5:**
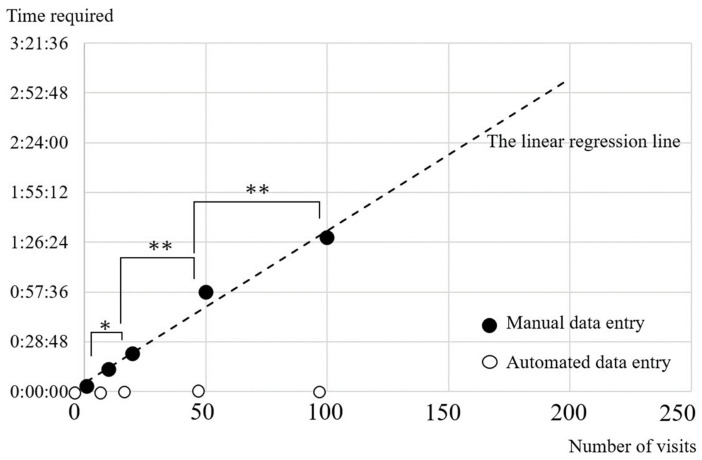
The linear regression graph shows the association between the number of visits (x) and the time required for manual or automated data entry (y). * *p* < 0.01, ** *p* < 0.001.

The mean Human Time by the three optometrists and the mean of the three AMD VIEWER Times (SD) were, respectively, as follows: 1 visit [3 min (m) 2 s (s) (41 s), 3.24 s (0.82 s)]; 10 visits [12 m 41 s (45 s), 2.66 s (0.43 s)]; 20 visits [22 m 0 s (2 m 40 s), 3.18 s (0.45 s)]; 50 visits [57 m 21 s (8 m 4 s), 2.83 s (0.16 s)]; and 100 visits [1 h 29 m 8 s (9 m 30 s), 3.00 s (0.11 s)]. The linear regression line calculated from the mean Human Time was y = 52(Second)x + 294(Second) (r = 0.99), and the estimated Human Time at 200 visits was 2 h 57 min 42 s. Multiple comparison test results showed that in Human Time, the time required for 20, 50, and 100 visits was significantly longer than that for the previous visits (10, 20, and 50 visits, respectively). On the other hand, there was no significant difference in AMD VIEWER time regardless of the number of visits (Steel–Dwass test) (Figure 5).

### 3.3. Doctors’ Recommendation

**Figure 6:**
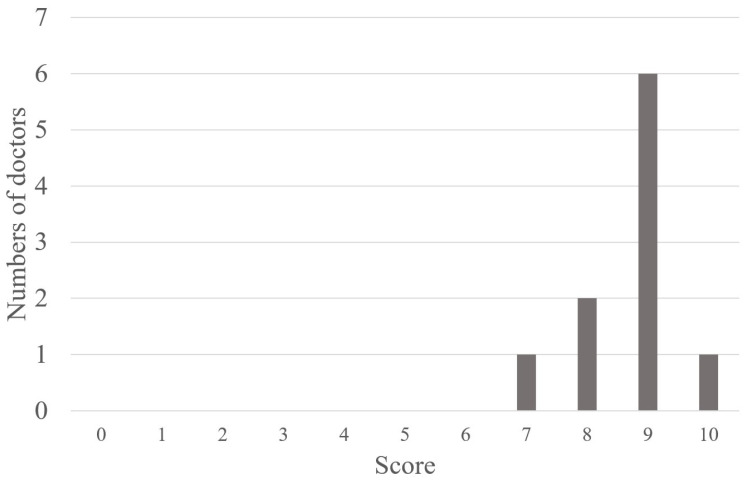
The bar graph shows the ophthalmologists’ recommendation score.

Of the ten ophthalmologists, none rated the recommendation score below 6, one rated it as 7, two rated it as 8, six rated it as 9, and one scored 10. As a result, in the classification for calculating the Net Promoter Score (NPS), 70% were Promoters, and 0% were Detractors, resulting in an NPS of 70% (Figure 6).

## 4. Discussion

### 4.1. Principal Results

Our development of AMD VIEWER facilitated an understanding of the long-term course of AMD. AMD patients over 5 years of hospital visits had an average of 45 visits and a maximum of 222 visits. In addition, 47.5% of the subjects dropped out, and the number of visits and treatments was significantly lower among dropout patients than among outpatients. As a result, their visual acuity was significantly lower than that of patients who continued their hospital visits. These results are consistent with previous reports [14,15] and strongly suggest the difficulty of maintaining long-term AMD treatment visits and the possibility that dropouts are not making appropriate medical and treatment decisions. Cho SC et al. reported that among 488 AMD patients (334 temporal neovascular AMD patients and 154 polypoidal choroidal vasculopathy patients), treatment discontinuation occurred in 66.0% of cases within 1.5 years after starting treatment [14]. Ramakrishnan MS and colleagues used data from the Comparison of Age-related Macular Degeneration Treatments Trials (CATT) to track 1178 patients over 2 years. The average number of missed appointments per patient was 2.4, and across all 4 indicators, patients who adhered most closely to regular examinations showed better visual outcomes. Missing just one examination resulted in a decrease of 0.7 in the letter score for vision [15].

Mutual recognition is a critical element in interpersonal communication [16]. For example, patients expect their doctors to remember the history of frequent vitreous injections. The average length of hospital visits for all patients with AMD included in this study was 1609 days (more than 4 years and 4 months), and the number of treatments was 7.5 (Table 1). A physician cannot memorize such frequent treatments over a long period for each patient, and the patients themselves do not always remember them accurately; AMD VIEWER can almost zero in on such gaps in recognizing the disease course. A lack of explanation and understanding of treatment effects contributes to poor compliance with anti-VEGF therapy for diabetic macular edema patients [17]. We believe there is a high need for AMD VIEWER, which allows physicians and patients to correctly update treatment efficacy to reduce the dropout rate of patients with age-related macular degeneration who require lifelong hospital visits [18].

In an examination of the efficiency of AMD VIEWER, human input required 57 min to obtain medical information for one eye of a patient with 50 visits. In contrast, AMD VIEWER displayed the same data in less than 4 s. Furthermore, the time required to input information increased significantly as hospital visits increased. Our linear regression model estimated that humans needed nearly 3 hours to input medical data for 200 visits. In contrast, AMD VIEWER did not experience display time changes even if the number of hospital visits increased. The extreme difference between the two time requirements means that AMD VIEWER’s high efficiency allows it to do what human input cannot in managing age-related macular degeneration.

The quality of decision making is directly proportional to the amount of information available [4], suggesting that data from AMD VIEWER contribute to good decision making. For example, even with similar OCT images and nearly equivalent visual acuity, the situation where AMD is initially diagnosed and after undergoing treatment numerous times are entirely different scenarios. Therefore, the significance of the AMD VIEWER, which allows for an instant overview of the long-term progression, is substantial. This is especially useful when a new physician takes over a patient’s care, particularly for those with a long history of treatment. We perceive the frequent change of physicians in long-term AMD clinical settings as a very realistic and significant issue. Furthermore, AMD VIEWER can determine disease trends by calculating simple regression lines of measured values. The method of accumulating large amounts and multiple aspects of ongoing medical data by information and communication technology (ICT) and analyzing them by statistical analysis is a major objective of the EMR system since it is not possible with human mental arithmetic [19]. Real-time sepsis warning systems using machine learning are already in practical use and have been reported to reduce the risk of death [20]. The regression line calculated by AMD VIEWER is similar to the regression line analysis of standard deviation values of glaucoma visual field tests [21] in estimating the prognosis of visual function for the target. Consequently, AMD VIEWER meets the high needs of patients and healthcare providers for predicting patient visual function. These facts are also demonstrated by the highly willing ophthalmologists who have used the product to recommend it to their close colleagues (NPS = 70, an NPS score of 50 or more is considered highly high product satisfaction) [12]. It has been reported that East Asians, including Japanese people, tend to choose midpoints rather than extremes on a given scale. [22] In this context, 7 out of 10 Japanese ophthalmologists rated the AMD VIEWER as a 9 or 10, qualifying as Promoters, suggesting strong support for the AMD VIEWER among ophthalmologists.

There is a risk that over reliance on mechanical systems can inadvertently lead to a dispersion of the user’s attention. At our facility, where the system has been implemented, we have established a rule where the examination begins by reviewing the patient’s current and previous visual acuity, OCT, and fundus photographs. Only after this initial review do we consult AMD VIEWER to understand the long-term progression. This rule is natural, based on the principle that the examining physician’s most immediate points of interest are the patient’s current visual acuity and OCT images. By adhering to this rule in using AMD VIEWER, we aim to prevent the dispersion of attention.

### 4.2. Limitations

This system is not commercially available but a prototype that we have created ourselves. Injection treatment information and vision test information are usually recorded in separate databases. To adapt the system to any facility, we need to digitize these data and build an application to link them. In addition, as is familiar to all ICTs, there are high costs associated with the system’s backbone that are not visible on the surface, such as countermeasures against network failures and recent malicious network attacks. The cost-effectiveness of system construction based on the effectiveness of AMD VIEWER in improving physician decision making and patient compliance is an essential issue for the future. In the long-term follow up of AMD patients with a very high rate of discontinuing outpatient visits, adopting an approach focused on preventing dropouts from the outset is crucial. We aim to address an important future task of developing a dropout prediction model using methods like Cox logistic regression based on clinical indicators such as age and visual acuity values, which are shown to influence dropout in this study. This model will then be integrated into AMD VIEWER.

## 5. Conclusions

It is impossible to collect the amount of information provided by AMD VIEWER in a long-term clinical study of AMD, and the ability of physicians and patients to share this information at each visit is likely to improve the quality of care and patient compliance. Although further research on the effectiveness of information presentation by AMD VIEWER for physicians and patients is needed, the results suggest that this system may be a powerful tool for supporting AMD treatment in clinical practice.

## Figures and Tables

**Table 1:** The details of each group and the demographic data.

	Total	Patients without Treatments	Patients with Treatments	Dropout Group	Active Group	*p*-Value (Dropout vs. Active)
Number	977	307	670 (including eight non-grouped patients ^a^)	315	347	
Age	76.1 (10.9)	71.6 (12.9)	78.1 (9.2)	79.0 (8.8)	77.3 (9.2)	*p* = 0.0124 by Tukey’s HSD test
Male (%)	703 (72.0%)	211 (68.7%)	492 (73.4%)	237 (75.2%)	248 (71.5%)	*p* = 0.292 by Fisher’s exact test
Observation period (days)	1609 (1457)	902 (1183)	1934 (1457)	1650 (1376)	2191 (1479)	*p* < 0.001 by Tukey’s HSD test
Consultations (times)	34.9 (34.4)	11.2 (14.4)	45.7 (35.5)	43.0 (35.5)	48.4 (35.7)	*p* = 0.0491 by Tukey’s HSD test
Treatments (times)	7.3 (11.7)	0	10.6 (12.8)	8.2 (9.3)	12.9 (14.5)	*p* = 0.0491 by Tukey’s HSD test
Minimum corrected visual acuity (LogMAR ^b^)	0.726 (0.787)	0.138 (0.395)	1.00 (0.78)	1.110 (0.782)	0.890 (0.762)	*p* < 0.001 by Steel–Dwass test

^a^ Among the eight non-grouped patients, four died during the follow-up period, and four were referred to other hospitals. ^b^ LogMAR is a logarithmic conversion of visual acuity that allows for statistical calculations. Higher values indicate poorer vision.

## Data Availability

Regarding the data, we are conducting analyses that include patients’ personal information. We will provide the data only after completing the necessary legal procedures within Japan and exclusively for the purpose of re-evaluating the scientific credibility.

## Supplementary Materials

The following supporting information can be downloaded at: https://www.mdpi.com/article/10.3390/bioengineering10121426/s1, Video S1: THE AMD VIEWER DEMO.

## Abbreviations

EMR: electronic medical record; AMD: age-related macular degeneration; VEGF: vascular endothelial growth factor; OCT: optical coherence tomography; PDT: photodynamic therapy; Main App: main application; ICT: Information and Communication Technology.
